# Brainstem Volume and White Matter Characteristics Associated with Cognitive Performance in Healthy Middle‐aged Adults

**DOI:** 10.1002/alz.093235

**Published:** 2025-01-09

**Authors:** Yanrong Li, Marie Caillaud, Isabelle Gallagher, Cherry Youn, Jessica Park, Andreana Haley

**Affiliations:** ^1^ The University of Texas at Austin, Austin, TX USA; ^2^ University of Texas at Austin, Austin, TX USA

## Abstract

**Background:**

Traditionally linked to essential physiological functions, the brainstem is now acknowledged for its role in cognition and dementia. Abnormal tau protein accumulation starts in the brainstem. Late life brainstem volume at baseline also predicts later Alzheimer's Disease conversion. Yet, brainstem's association with cognitive function in healthy middle‐aged adults has been insufficiently studied. This research investigates the brainstem’s structure, focusing on volume and white matter integrity, and their correlation with cognitive performance in this healthy middle‐aged adults.

**Method:**

A total of 202 middle‐aged adults aged 40 to 60, free of clinical conditions were enrolled. They underwent a comprehensive neuropsychological assessment evaluating their executive function, attention, and memory performance. T1 and diffusion tensor images (DTI) were collected on a 3T scanner. Brainstem and total intracranial volumes (TIV) were extracted from the T1 images. Fractional anisotropy (FA) values of superior and middle cerebellar puncle were extracted from the DTI images.

**Result:**

After controlling for age, sex, education, and total intracranial volume, larger brainstem volume is significantly associated with better memory performance (F(5, 196) = 6.185, R^2^ = 0.1363, P < .001; β_brainstem vol_ = .069, P = .025) but not attention or executive function. Additionally, APOE4 carrier status significantly moderates the correlation between brainstem volume and attention (F(7, 85) = 2.902, R^2^ = 0.193, P < .01; β_brainstem vol:APOE4 status_ = .131, P = .034). After controlling for age, sex, and education, higher superior cerebellum peduncle FA value is significantly associated with better memory (F(4, 128) = 6.58, R^2^ = 0.1706, P < .001; β_SCP FA_= 5.098, P = .0018), attention (F(4, 128) = 4.706, R^2^ = 0.13, P = .001; β_SCP FA_= 3.983, P = .009), and executive function (F(4, 128) = 2.963, R^2^ = 0.085, P = .022; β_SCP FA_= 3.945, P = .018).

**Conclusion:**

The study highlights the brainstem's significant association with memory performance and attention in middle‐aged adults, particularly among APOE4 carriers. This emphasizes the brainstem's potential as an early indicator of cognitive decline. The significant correlation between brainstem FA values and cognitions suggests that further exploration of other brainstem's measures should be included in future studies.

